# Supplementary material for: Ribonucleoside Hydrolases–Structure, Functions, Physiological Role and Practical Uses
Source: Biomolecules. 2023 Sep 12;13(9):1375. doi: 10.3390/biom13091375 (PMC10526354; doi:10.3390/biom13091375)
Supplement: Supplementary file 1 [file biomolecules-13-01375-s001.zip › Figure S1.pdf]

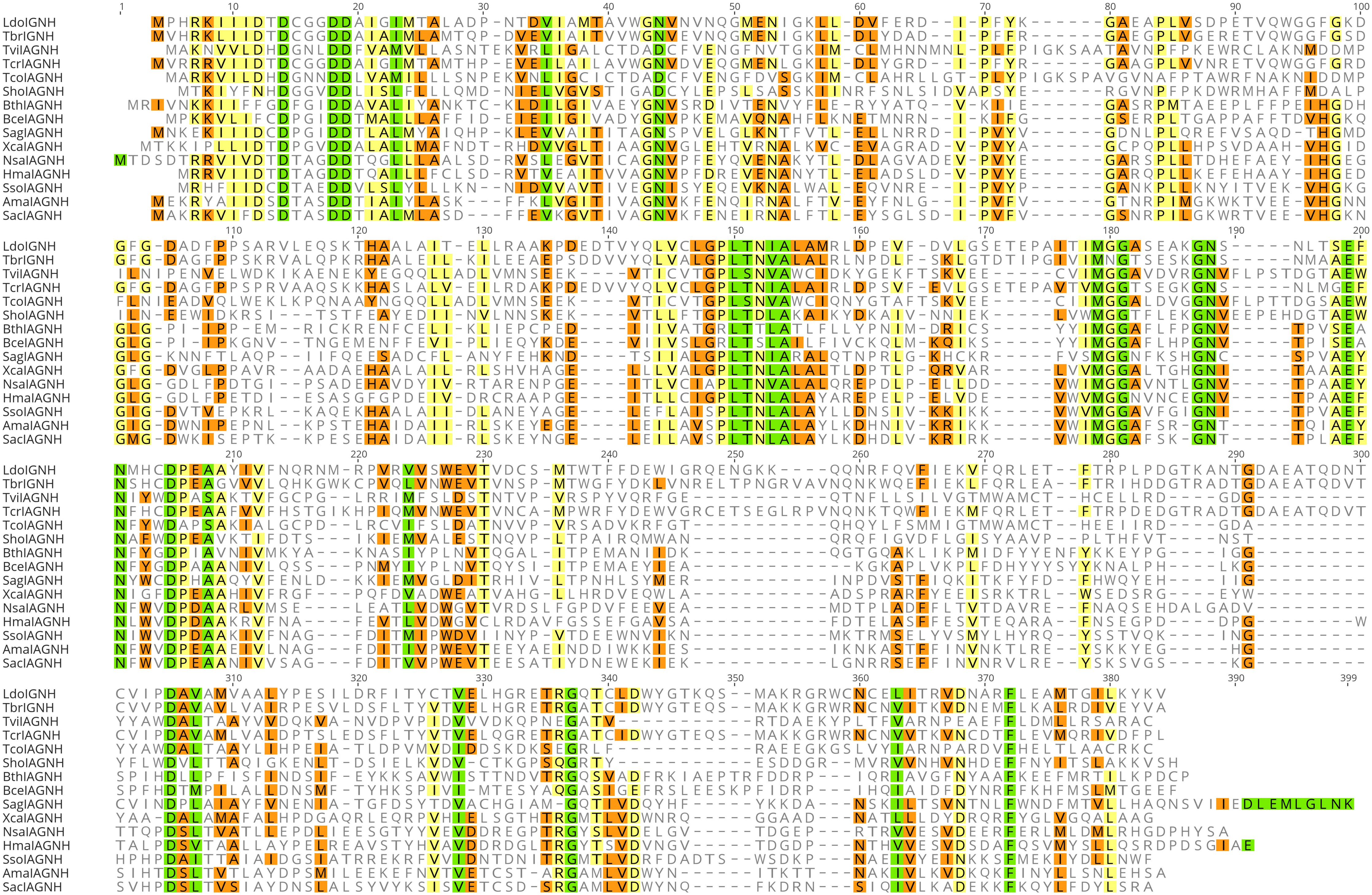

LdoIGNH
TbrIGNH
TviIAGNH
TcrIAGNH
TcoIAGNH
ShoIAGNH
BthIAGNH
BceIAGNH
SagIAGNH
XcaIAGNH
NsaIAGNH
HmaIAGNH
SsoIAGNH
AmaIAGNH
SacIAGNH
