# Supplementary material for: Ribonucleoside Hydrolases–Structure, Functions, Physiological Role and Practical Uses
Source: Biomolecules. 2023 Sep 12;13(9):1375. doi: 10.3390/biom13091375 (PMC10526354; doi:10.3390/biom13091375)
Supplement: Supplementary file 1 [file biomolecules-13-01375-s001.zip › Figure S2.pdf]

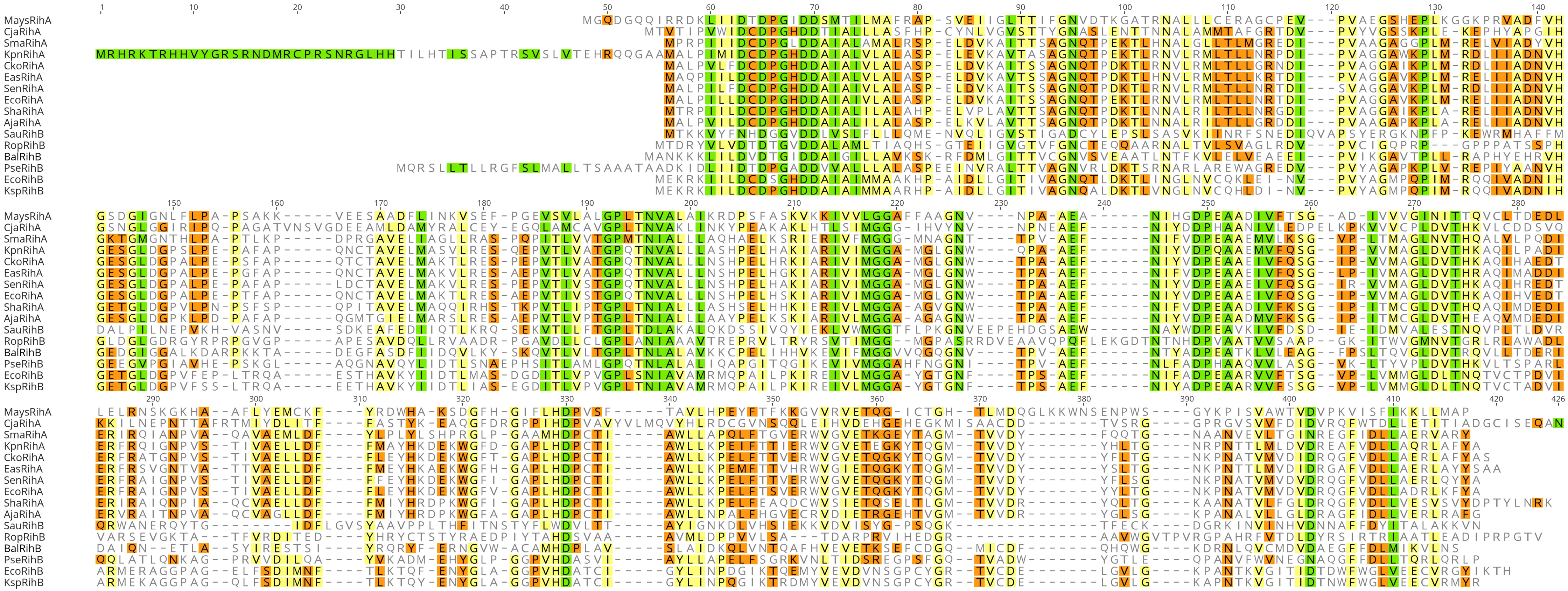
MaysRihA
CjaRihA
SmaRihA
KpnRihA
CkoRihA
EasRihA
SenRihA
EcoRihA
ShaRihA
AjaRihA
SauRihB
RopRihB
BalRihB
PseRihB
EcoRihB
KspRihB
